# Barriers to survivorship care for adolescent and young adult cancer survivors: perspectives of survivors and cancer specialists

**DOI:** 10.3389/fonc.2026.1977660

**Published:** 2026-10-14

**Authors:** Yuki Kojima, Yayoi Ando, Yuki Nogami, Shintaro Iwata, Tatsuya Suzuki, Eriko Satomi

**Affiliations:** 1 Department of Medical Oncology, National Cancer Center Hospital, Tokyo, Japan; 2 Clinical Research Support Office, National Cancer Center Hospital, Tokyo, Japan; 3 Department of Pediatric Oncology, National Cancer Center Hospital, Tokyo, Japan; 4 Department of Musculoskeletal Oncology and Rehabilitation, National Cancer Center Hospital, Tokyo, Japan; 5 Department of Hematology , National Cancer Center Hospital, Tokyo, Japan; 6 Department of Palliative Medicine, National Cancer Center Hospital, Tokyo, Japan

**Keywords:** adolescent and young adult, cancer survivorship, long-term follow-up, survivorship care, telemedicine

## Abstract

**Background:**

Adolescent and young adult (AYA) cancer survivors have complex long-term survivorship needs beyond cancer recurrence surveillance. Although survivor concerns and healthcare provider perspectives have been reported separately, few studies have evaluated both within the same healthcare setting. We conducted complementary surveys of AYA cancer survivors and physicians to characterize survivorship concerns, barriers to long-term follow-up (LTFU), and telemedicine preferences.

**Methods:**

This single-center, cross-sectional study included questionnaire surveys of 98 AYA cancer survivors receiving outpatient follow-up after cancer treatment and 45 physicians involved in AYA survivorship care at the National Cancer Center Hospital, Japan. Survivors were surveyed regarding LTFU, survivorship concerns, and telemedicine preferences, and physicians regarding LTFU practices, referral barriers, and AYA-specific challenges. Associations between survivor characteristics and telemedicine preference were explored using univariable analyses.

**Results:**

Among survivors, 84 of 98 (85.7%) considered LTFU necessary, and 51 (52.0%) reported difficulty attending follow-up because of school or work commitments. Of 91 survivors evaluable for telemedicine preference, 69 (75.8%) were likely or somewhat likely to use at least one telemedicine service. Among all 98 survivors, frequently selected telemedicine topics included disease information (45.9%), daily activities (31.6%), anxiety and worry (30.6% each), financial issues (29.6%), and work- or school-related concerns (27.6%). No demographic, clinical, treatment-related or healthcare utilization factors were significantly associated with telemedicine preference. Among physicians, 13 of 44 (29.5%) reported an established departmental LTFU policy. Follow-up at the treating cancer center was supported by 76.2% for survivors at high risk of recurrence but by only 21.4% for all AYA survivors. Major barriers to coordinated care included difficulty identifying hospitals providing cancer-specific LTFU (73.8%) or multidisciplinary care (71.4%). Physicians identified reproductive medicine and life events as major AYA-specific challenges and mental health, endocrine disorders, and sexual and reproductive health as areas requiring additional support.

**Conclusions:**

AYA cancer survivors reported substantial survivorship concerns, whereas physicians identified barriers to coordinated multidisciplinary care. Telemedicine was of interest to many survivors and warrants prospective evaluation as a potential component of multidisciplinary survivorship care.

## 1 Introduction

Adolescents and young adults (AYAs), defined as individuals aged 15–39 years, differ from both pediatric and older adult populations in their biology, treatment profiles, and psychosocial circumstances (1–3). The number of AYA cancer survivors continues to increase worldwide due to advances in cancer diagnosis and treatment (2, 4). As a result, greater attention is now directed toward preventing and managing late effects and maintaining long-term quality of life in this population (5). In Japan, approximately 20, 000 AYAs are newly diagnosed with cancer annually, and the growing survivor population presents challenges to the delivery of long-term survivorship care that parallel those encountered internationally (1).

Current clinical practice guidelines emphasize that the needs of AYA cancer survivors extend far beyond recurrence surveillance and include fertility preservation and reproductive health, psychosocial support, educational and vocational assistance, health promotion, and guidance regarding major life transitions, such as intimate relationships, marriage, pregnancy, and career development (5). These multidimensional needs require coordinated, multidisciplinary care throughout survivorship.

Although these needs are now widely acknowledged, survivorship care for AYAs remains inconsistent and poorly standardized in most settings (2). In pediatric oncology, structured risk-based long-term follow-up programs, exemplified by the Children’s Oncology Group (COG) Long-Term Follow-Up Guidelines, provide comprehensive recommendations for surveillance, prevention, and management of treatment-related late effects (6). In contrast, structured survivorship care pathways for AYA cancer survivors are less well-established.

Digital health strategies, including telemedicine, have been proposed as approaches to improve access to survivorship care, particularly for survivors facing geographic, occupational, or educational barriers to long-term follow-up. Recent reviews suggest that telemedicine can effectively support symptom management, psychosocial care, and health information exchange, although evidence regarding its role in routine survivorship surveillance remains limited, and implementation challenges persist (7, 8). Because AYA survivors face developmental and social circumstances that differ from those of older individuals, their telemedicine preferences deserve specific study.

Several studies have investigated the experiences and unmet needs of AYA cancer survivors, whereas others have explored healthcare providers’ perspectives on the delivery of survivorship care or the implementation of telemedicine (9–11). However, these perspectives have largely been evaluated independently, and few studies have assessed survivor concerns and healthcare provider perspectives in the same healthcare setting. Without data from both groups, gaps between what survivors need and what clinicians provide are likely to go unrecognized. Therefore, we conducted complementary questionnaire surveys among AYA cancer survivors and specialists at a comprehensive cancer center in Japan to characterize survivorship concerns and barriers to long-term follow-up from both survivor and physician perspectives and to explore survivors’ preferences for telemedicine.

## 2 Methods

### 2.1 Study design

This single-center, cross-sectional study consisted of two complementary questionnaire surveys conducted at the National Cancer Center Hospital, Tokyo, Japan. The first survey targeted AYA cancer survivors to evaluate their experiences, survivorship concerns, and preferences during LTFU. The second survey targeted physicians involved in the care of AYA cancer survivors to assess their current survivorship care practices and perceived barriers to LTFU. The physician questionnaire was developed after completion of the survivor survey and informed by the findings from the survivor survey, so that the two surveys would address survivorship care from both the survivor and physician perspective. Throughout this study, AYA was defined as individuals aged 15–39 years. This study was conducted in accordance with the STROBE (Strengthening the Reporting of Observational Studies in Epidemiology) guidelines.

### 2.2 Survey instruments

Two original questionnaires were developed by the study investigators: one for AYA cancer survivors and another for physicians involved in the care of AYA cancer survivors. The survivor questionnaire assessed demographic characteristics, treatment history, current follow-up practices, perceptions of long-term follow-up, and attitudes toward telemedicine. The physician questionnaire evaluated institutional practices, perceived challenges in long-term follow-up, multidisciplinary care needs, and telemedicine. English translations of both questionnaires are provided in **Supplementary Appendices 1** and **2**.

Both questionnaires were developed specifically for this exploratory study by all six study investigators, including pediatricians, medical oncologists, and surgeons involved in the care of AYA patients, some of whom also provide dedicated survivorship care. Questionnaire items were generated based on the investigators’ clinical experience and a review of the relevant literature. Survivors were not involved in questionnaire development. Cognitive interviewing, pilot testing, and formal assessment of content validity were not performed. Both questionnaires were administered in Japanese. The English versions provided in the **Supplementary Material** were translated from the original Japanese questionnaires by the authors.

### 2.3 Survivor survey

Cancer survivors aged 15–39 years receiving follow-up care at the National Cancer Center Hospital were invited to participate in an anonymous, cross-sectional questionnaire survey between December 10, 2021, and March 31, 2022. Eligible participants were aged 15–39 years at the time of survey participation, had achieved remission, received no active anticancer treatment, and were undergoing outpatient follow-up in one of five departments (Pediatric Oncology, Bone and Soft Tissue Oncology, Medical Oncology, Hematology, or Hematopoietic Stem Cell Transplantation). The age criterion was based on age at survey participation rather than age at cancer diagnosis; therefore, the study population included some survivors who had been diagnosed with cancer before the age of 15. Patients considered inappropriate for participation by the investigators were excluded.

Potential participants received written information about the study during their outpatient visits. Participants completed an anonymous, self-administered questionnaire, either electronically using a QR code or on paper. The questionnaire collected information on demographic characteristics, educational and employment status, cancer diagnosis and treatment history, healthcare utilization, awareness of LTFU, preferences regarding follow-up care, attitudes toward telemedicine, and survivorship-related concerns. Of the 155 questionnaires distributed, 98 were returned (response rate: 63.2%), including 56 web-based and 42 paper-based questionnaires.

### 2.4 Physician survey

Following completion of the survivor survey, a cross-sectional questionnaire survey was conducted among physicians involved in the care of AYA cancer survivors between October 11 and October 17, 2023. Physicians from ten clinical departments that routinely care for AYA cancer survivors were invited to participate.

The questionnaire assessed physician characteristics, current LTFU practices, preferred models of survivorship care, referral patterns for LTFU, barriers to collaboration with community healthcare providers, perceived challenges unique to AYA survivorship care, and opinions regarding the implementation of telemedicine follow-up services. Several items were informed by findings from the survivor survey to examine physicians’ perspectives on issues identified by survivors. Of the 64 invited physicians, 45 responded (response rate: 70.3%).

### 2.5 Outcomes

The primary objective was to characterize the concerns and preferences of AYA cancer survivors regarding LTFU and to describe current LTFU practices and perceived barriers among physicians involved in AYA survivorship care. The secondary objectives were to describe survivors’ preferences regarding telemedicine and preferred topics for telemedicine consultation, and to explore associations between telemedicine preference and survivor characteristics.

Preference for telemedicine was defined as selecting “likely” or “somewhat likely” for at least one of three services: an online visit with a physician, an online consultation with a physician, or an online consultation with a non-physician healthcare professional. Participants who completed all three items and selected “not likely” or “not at all likely” for all three services were classified as not preferring telemedicine. Participants with incomplete responses to any of the three items were excluded from the analysis of telemedicine preferences.

### 2.6 Statistical analysis

No formal sample size calculation was performed because of the exploratory nature of the study. Descriptive statistics were used to summarize the responses to the survivor and physician surveys. Associations between telemedicine preference and survivor characteristics were evaluated using univariable analyses. Continuous variables were summarized as medians with interquartile ranges (IQRs) and compared using the Mann–Whitney *U* test. Categorical variables were summarized as numbers and percentages and compared using Fisher’s exact test. Missing data were not imputed, and analyses were performed using available data. All tests were two-sided, and P values <0.05 were considered statistically significant. Statistical analyses were performed using GraphPad Prism, version 11 (GraphPad Software, San Diego, CA, USA).

### 2.7 Ethics

The survivor survey was approved by the Institutional Review Board of the National Cancer Center, Tokyo, Japan (Approval No. 2021-309). Participants received written information about the study, and completion of the anonymous questionnaire was regarded as consent to participate. The physician survey was an anonymous survey of healthcare professionals that did not involve patient information or medical record review and did not require institutional review board approval in accordance with institutional policy.

## 3 Results

### 3.1 Survivor characteristics

A total of 98 AYA cancer survivors completed the questionnaire. Their demographic and clinical characteristics are summarized in **Supplementary Table S1**. The median age at survey participation was 29 years (range, 15–39), and the median age at cancer diagnosis was 25 years (range, 0–38). Twelve participants (12.2%) had been diagnosed before the age of 15. At the time of the survey, 42 (42.9%) participants were in their 20s and 45 (45.9%) were in their 30s. The median time since completion of cancer treatment was 3.7 years. Nearly half of the participants were regular employees (49.0%), and 80.0% had received chemotherapy.

### 3.2 Current survivorship care patterns

Most survivors (84/98, 85.7%) considered LTFU necessary after completion of cancer treatment, with surveillance for cancer recurrence being the most frequently reported reason (**Figure 1**). In an exploratory analysis stratified by time since treatment completion, 66 of 77 survivors (85.7%) who were <5 years from treatment completion considered LTFU necessary, compared with 18 of 21 (85.7%) who were ≥5 years from treatment completion (Fisher’s exact test, p = 1.000). Twenty-seven survivors (27.6%) were followed up in more than one clinical department, and 26 (26.5%) received shared follow-up at another hospital in addition to the National Cancer Center Hospital. Fifty-one survivors (52.0%) reported that school or work commitments interfered with their attendance at regular follow-up visits, and 28 (28.6%) required more than 60 min to travel to the hospital.

**Figure 1:**
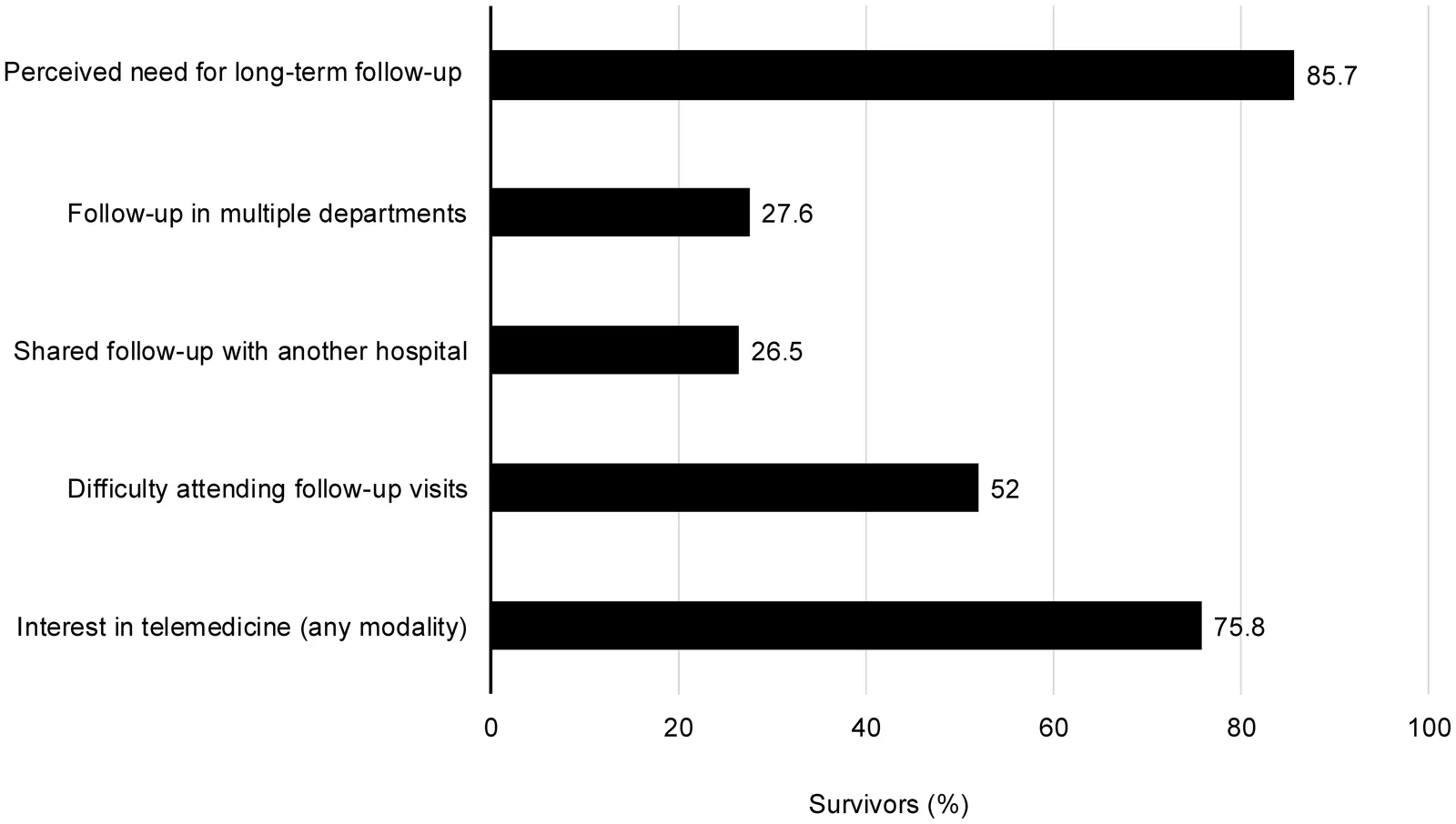
Long-term follow-up experiences and perceived needs among AYA cancer survivors. Responses of 98 AYA cancer survivors regarding long-term follow-up (LTFU) after completion of cancer treatment. The figure shows the proportion of survivors who considered LTFU necessary, received follow-up in multiple clinical departments, received shared follow-up with another hospital, and reported difficulty attending follow-up visits because of school or work commitments.

### 3.3 Preferred topics for telemedicine

Survivors were asked to select topics they preferred to discuss through telemedicine (**Figure 2**). The most frequently selected topic was disease information (45.9%), followed by daily activities (31.6%), anxiety and worry (30.6% each), financial issues (29.6%), and work- and school-related concerns (27.6%). Depression (24.4%), childbearing (21.4%), and pain (21.4%) were also frequently selected.

**Figure 2:**
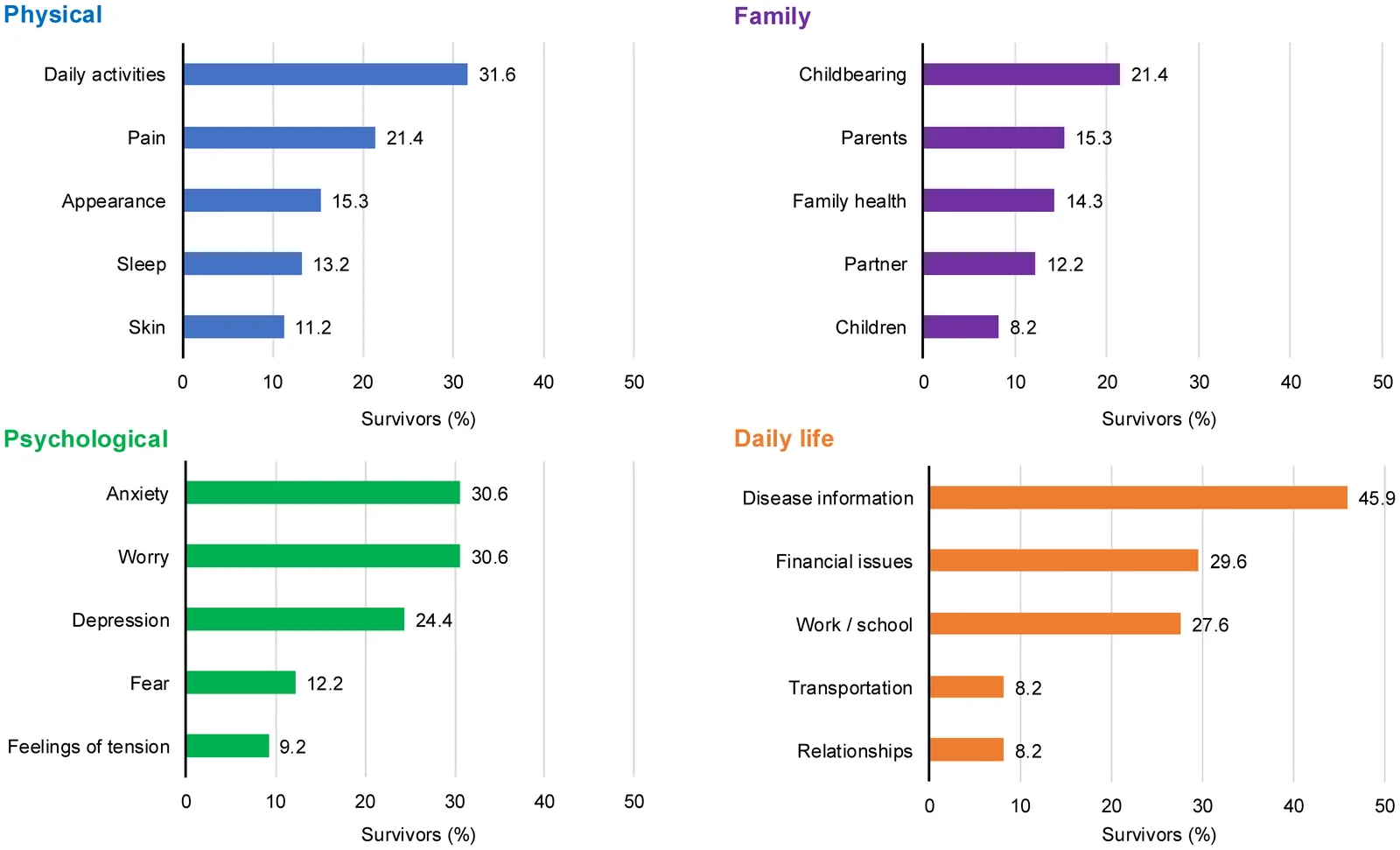
Preferred topics for telemedicine among AYA cancer survivors. Survivors selected topics that they preferred to discuss using telemedicine during long-term follow-up. Results are presented by domain (physical, psychological, family, and daily life), with the five most frequently selected topics shown in each category. Multiple responses were permitted.

### 3.4 Survivor characteristics according to telemedicine preference

Among the 91 survivors who completed all three telemedicine-related items, 69 (75.8%) indicated that they were likely or somewhat likely to use at least one telemedicine service, whereas 22 (24.2%) did not indicate a preference for any of the three services. Comparisons of participant characteristics according to telemedicine preference are shown in **Table 1**. Telemedicine preference was not significantly associated with age at survey, sex, age at cancer diagnosis, time since treatment completion, treatment exposures, travel time, transportation cost, school- or work-related time constraints, follow-up in multiple clinical departments, or follow-up in multiple hospitals (all P > 0.05; **Table 1**).

**Table 1:** Comparison of participant characteristics according to telemedicine preference.

Characteristic	Preferred telemedicine	Did not prefer telemedicine	P value
Age at survey, years	29 (24–36)	31 (25.8–37)	0.425
Female, n (%)	36 (52.2%)	8 (36.4%)	0.228
Age at cancer diagnosis, years	25 (16.3-30.2)	26 (18-34.5)	0.481
Time since treatment completion, years	2.7 (1.0-4.95)	1.25 (0.68-3.475)	0.121
Chemotherapy, n (%)	57 (82.6%)	20 (95.2%)	0.285
Radiotherapy, n (%)	26 (37.7%)	9 (42.9%)	0.799
Surgery, n (%)	31 (44.9%)	10 (47.6%)	>0.999
Hematopoietic stem cell transplantation, n (%)	11 (15.9%)	3 (14.3%)	>0.999
One-way travel time, min	60 (40–80)	55 (37.5–90)	0.799
One-way transportation cost, JPY^†^	600 (325–1000)	578 (400–1400)	0.610
Time constraints, n (%)	37 (53.6%)	14 (63.6%)	0.467
Multiple departments, n (%)	21 (30.9%)	6 (27.3%)	>0.999
Multiple hospitals, n (%)	22 (31.9%)	3 (13.6%)	0.109

Data are presented as median (interquartile range) or n (%). Continuous variables were compared using the Mann–Whitney U test and categorical variables using Fisher’s exact test. Analyses were based on available cases, and missing data were not imputed. Telemedicine preference was evaluated among 91 survivors who completed all three telemedicine preference items.

^†^ Transportation cost had missing values.

### 3.5 Physician perspectives on survivorship care

A total of 64 physicians from 10 clinical departments were invited, of whom 45 unique physicians completed the survey (response rate: 70.3%). One respondent reported affiliation with both Medical Oncology and Urology/Retroperitoneal Oncology; therefore, the sum of department-specific responses was 46. Seventeen respondents (37.8%) had more than 20 years of clinical experience, 12 (26.7%) had 15–20 years, 15 (33.3%) had 10–15 years, and one (2.2%) had less than 10 years (**Supplementary Table S2**). Item-level response rates varied slightly across questionnaire sections; denominators for individual items are reported below and reflect the number of physicians who answered each specific question.

Among the 44 physicians who responded to the item regarding departmental LTFU policy, 13 (29.5%) reported that their department had an established LTFU policy for all patients. Responses were not fully consistent among physicians within some departments; therefore, these findings represent individual physicians’ reports of departmental practice rather than independently verified department-level policies. Twenty-seven (61.4%) indicated that LTFU was primarily provided at the National Cancer Center Hospital (**Figure 3A**). Among the 42 physicians who responded to the question regarding preferred models of follow-up, 32 (76.2%) considered that patients at a high risk of recurrence should continue follow-up at the treating cancer center. Twenty-two physicians (52.4%) supported continued follow-up at the treating cancer center for patients requiring management of treatment-related complications or toxicities, 21 (50.0%) for those at a high risk of late effects, and nine (21.4%) for all AYA cancer survivors.

**Figure 3:**
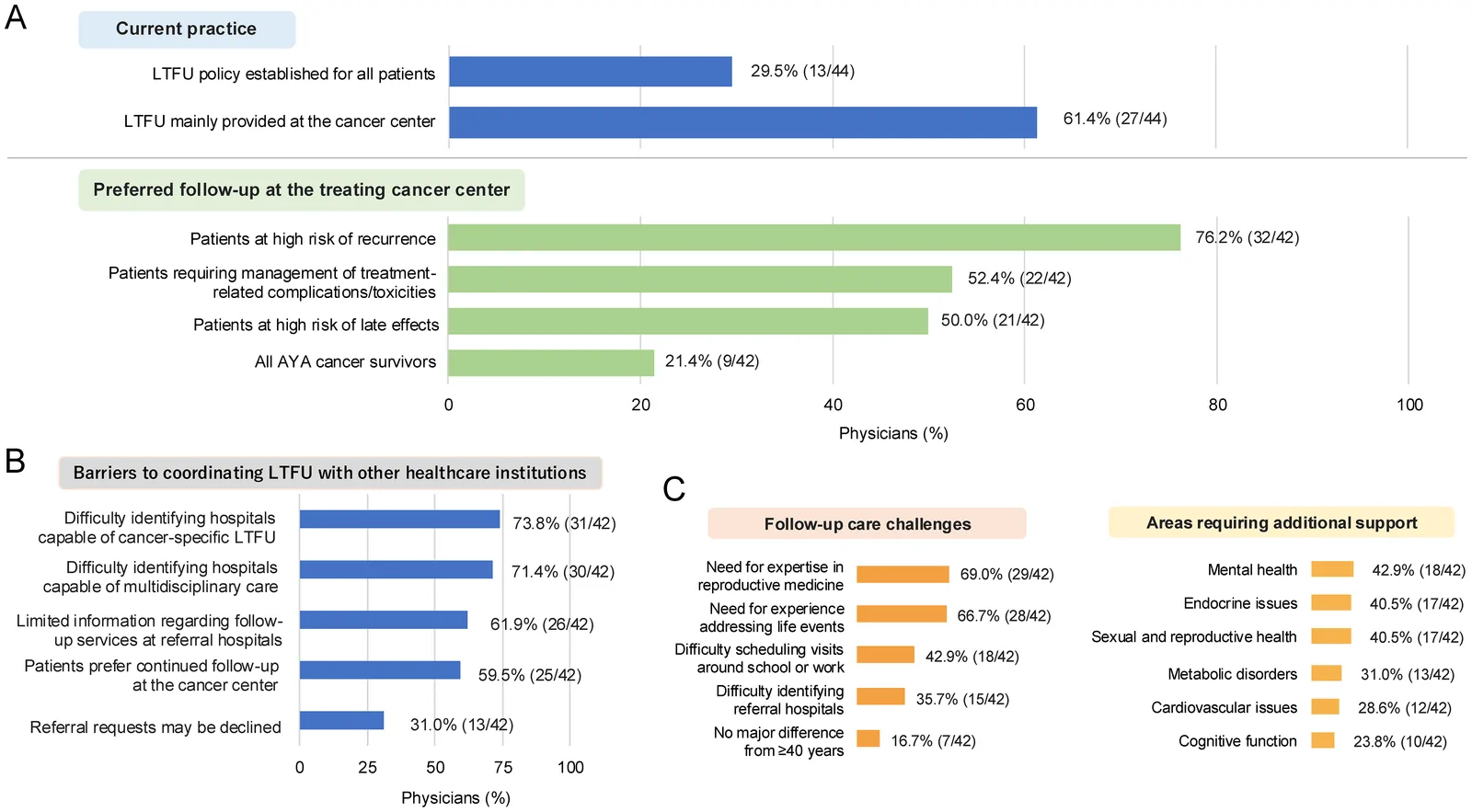
Physician perspectives on long-term survivorship care for AYA cancer survivors. Physicians were surveyed regarding current and preferred models of long-term survivorship care for AYA cancer survivors. Panel **(A)** shows current practices and preferred models for long-term follow-up. Responses regarding departmental LTFU policies represent individual physicians’ reports and were not independently verified at the department level. Panel **(B)** summarizes barriers to coordinating long-term follow-up with other healthcare institutions. Panel **(C)** presents challenges specific to AYA survivorship care, including follow-up care needs and clinical areas requiring additional support. Multiple responses were permitted for Panels **(A–C)**, except for the two current-practice items in Panel **(A)**. Percentages were based on the number of respondents to each question.

Physicians reported several barriers to coordinating LTFU with other healthcare institutions (**Figure 3B**). The most frequently reported barriers were difficulty in identifying hospitals capable of providing cancer-specific LTFU (31/42, 73.8%) or multidisciplinary care (30/42, 71.4%), limited information regarding follow-up services at referral hospitals (26/42, 61.9%), and patient preference for continued follow-up at the treating cancer center (25/42, 59.5%).

Regarding challenges specific to AYA survivorship care, 29 of the 42 physicians (69.0%) reported a need for expertise in reproductive medicine, and 28 (66.7%) reported a need for experience in addressing life events (**Figure 3C**). Mental health (18/42, 42.9%), endocrine issues (17/42, 40.5%), and sexual and reproductive health (17/42, 40.5%) were the clinical areas most frequently identified as requiring additional support.

## 4 Discussion

In this study, we surveyed both AYA cancer survivors and physicians at a single cancer center to examine survivorship care from two complementary viewpoints. Three principal findings were identified. First, most survivors considered long-term follow-up necessary after completion of cancer treatment, whereas physicians most frequently supported continued follow-up at the treating cancer center for survivors at high risk of recurrence, treatment-related toxicities, or late effects; relatively few supported such follow-up for all AYA survivors. Second, more than half of survivors reported that school or work commitments interfered with attendance at follow-up visits, a practical barrier that survivorship programs should account for. Third, the two surveys identified different areas of emphasis regarding survivorship care, highlighting the importance of coordinated multidisciplinary survivorship care, while many survivors expressed interest in telemedicine as a potential component of survivorship care.

In the present study, most survivors considered LTFU necessary after completion of active cancer treatment. This finding should be interpreted in light of the relatively short median interval of 3.7 years since treatment completion, as recurrence surveillance may still have been an important component of ongoing follow-up for many participants. However, in an exploratory analysis stratified by time since treatment completion, the proportion of survivors who considered LTFU necessary was identical among those <5 years and ≥5 years from treatment completion (85.7% in both groups). Although the number of survivors ≥5 years from treatment completion was relatively small, this finding suggests that the perceived need for LTFU may persist beyond the earlier post-treatment period. More than half of the participants reported difficulty attending follow-up visits because of school or work commitments, and more than one-quarter required over an hour to travel to the cancer center. Barriers to accessing survivorship care include competing educational and occupational obligations, geographic and logistical constraints, and uncertainty regarding who is responsible for long-term surveillance (2, 12). These barriers may be particularly relevant for AYA survivors, who are often pursuing education, entering the workforce, or starting families, making it difficult to sustain conventional hospital-based daytime follow-up over extended periods.

Physicians’ responses varied according to survivors’ clinical circumstances, with continued follow-up at the treating cancer center most frequently supported for those at a high risk of recurrence, treatment-related toxicities, or late effects. Comprehensive AYA survivorship care requires competencies that extend beyond those of conventional oncological surveillance. Oncologists frequently lack confidence and accurate knowledge regarding fertility preservation options, costs, and timing (13), and formal training in sexual and reproductive health remains inconsistently incorporated into hematology-oncology fellowship curricula (14). Similarly, addressing psychological distress and late endocrine effects within the time constraints of routine oncology practice is widely reported as difficult, suggesting that specialists face barriers related to training, clinic time, and institutional infrastructure when attempting to deliver comprehensive survivorship care (5, 13, 14).

Digital health strategies, including telehealth and web-based platforms, have been proposed as approaches to facilitating access to survivorship care, particularly for survivors constrained by distance, work, or school commitments (15). In our study, approximately three-quarters of survivors expressed interest in at least one telemedicine service. Of particular interest, the telemedicine topics survivors selected were not limited to disease-related questions but included financial issues, work- or school-related concerns, and psychological concerns, suggesting that survivors may be interested in using telemedicine to discuss a broad range of survivorship-related issues. In exploratory analyses, telemedicine preference was not significantly associated with demographic, cancer-related, treatment-related, or logistical factors. Given the relatively small sample size, these findings should be interpreted cautiously, and larger studies are needed to identify which survivors are most likely to prefer telemedicine and to prospectively evaluate its role in survivorship care. However, previous studies have suggested a gap between provider and survivor attitudes toward telehealth; providers have generally reported greater acceptance of remote survivorship care than survivors themselves, suggesting that digital interventions should be designed according to survivors’ actual preferences rather than assumptions regarding their needs (16).

Although survivor-reported barriers and specialist-reported challenges have been described separately in the literature, few studies have examined both perspectives simultaneously within the same institutional context, and data specific to the Japanese healthcare setting remain particularly scarce. To the best of our knowledge, this is one of the few studies to evaluate the complementary perspectives of AYA cancer survivors and physicians within the same institutional setting. Recognizing where survivor-reported concerns align with and diverge from physician perspectives can help guide the design of survivorship care that is both practical for institutions and responsive to survivors.

This study had several limitations. First, it was conducted at a single institution with a relatively small sample size, which may limit its generalizability. Second, the questionnaires were developed specifically for this study and have not been formally validated. Third, the survivor survey was conducted between December 2021 and March 2022, during the COVID-19 pandemic, and the physician survey was conducted approximately 18 months later, in October 2023. Telemedicine availability, clinical practice, and patient attitudes may have changed both during this interval and since the surveys were conducted. Therefore, particularly with respect to telemedicine, our findings should be interpreted as reflecting the healthcare context of 2021–2023 and may not fully represent current practice or preferences. Moreover, the survey assessed hypothetical preferences for telemedicine and did not evaluate the implementation, feasibility, or effectiveness of a telemedicine intervention. Fourth, the survivor survey included only individuals who were attending outpatient follow-up at our institution. Survivors who had discontinued follow-up, transferred their care, or were unable to attend because of geographic, occupational, financial, or other barriers were not represented. Such individuals may experience different or greater barriers to survivorship care; therefore, barriers to follow-up may have been underestimated in our study, and the findings should not be generalized to all AYA cancer survivors. Finally, only physicians were included in the provider survey; the perspectives of nurses and other allied health professionals were not evaluated.

Our findings highlight the importance of incorporating both survivor-reported concerns and physician perspectives into the design of survivorship care for AYA cancer survivors. Telemedicine was of interest to many survivors and warrants prospective evaluation as a potential component of multidisciplinary survivorship care.

## Data Availability

The raw data supporting the conclusions of this article will be made available by the authors, without undue reservation.
