# Supplementary material for: Barriers to survivorship care for adolescent and young adult cancer survivors: perspectives of survivors and cancer specialists
Source: Front Oncol. 2026 Oct 14;16:1977660. doi: 10.3389/fonc.2026.1977660 (PMC13713312; doi:10.3389/fonc.2026.1977660)
Supplement: Supplementary file 1 [file DataSheet1.docx]

**Supplementary Appendix 1**

**English Translation of the AYA Cancer Survivor Long-term Follow-up Questionnaire**

***Section I. Participant Characteristics***

**1. About yourself**

**1.1 Sex**

□ Male

□ Female

□ Prefer not to answer

**1.2 Age**

Current age: ______ years

Age at cancer diagnosis: ______ years

Age at completion of cancer treatment: ______ years

Completion of treatment: Year ______, Month ______

**1.3 Cancer diagnosis:** __________

**1.4 Department(s) where you received cancer treatment**

**Please select all departments where you received cancer treatment (select all that apply).**

□ Neurosurgery and Neuro-Oncology

□ Head and Neck Surgery

□ Plastic and Reconstructive Surgery

□ Medical Oncology

□ Thoracic Oncology

□ Gastric Surgery

□ Gastrointestinal Medical Oncology

□ Respiratory Endoscopy

□ Hepatobiliary and Pancreatic Oncology

□ Gynecology

□ Dermatologic Oncology

□ Hematopoietic Stem Cell Transplantation

□ Pediatric Surgery

□ Dentistry

□ Palliative Medicine

□ Diagnostic Radiology

□ Diagnostic Pathology

□ Experimental Therapeutics

□ Ophthalmic Oncology

□ Head and Neck and Esophageal Medical Oncology

□ Breast Surgery

□ Thoracic Surgery

□ Esophageal Surgery

□ Colorectal Surgery

□ Gastrointestinal Endoscopy

□ Hepatobiliary and Pancreatic Surgery

□ Urology

□ Musculoskeletal Oncology and Rehabilitation

□ Hematology

□ Pediatric Oncology

□ General Internal Medicine

□ Anesthesiology and Intensive Care Medicine

□ Psycho-Oncology

□ Radiation Oncology

□ Laboratory Medicine

**2. Current living situation**

**2.1 Residence**

Current prefecture of residence:　 ______

Travel time to this hospital (one way):　______ minutes

Transportation cost (one way):　JPY________

**2.2 Current educational or employment status**

Please select all that apply.

□ Junior high school student

□ High school student

□ Vocational school student

□ University / Graduate school student

□ Full-time employee

□ Self-employed

□ Part-time employee

□ Unemployed

□ Homemaker

□ Other (__________________)

***Section II. Cancer Treatment and Treatment-related Complications***

In this questionnaire, “cancer treatment” refers only to treatment directed at the cancer itself and does not include treatment for late effects or long-term complications.

**1. Initial cancer treatment**

Please select all treatments you received during your initial cancer treatment.

□ Surgery

□ Systemic drug therapy (including endocrine therapy)

□ Radiation therapy

□ Autologous hematopoietic stem cell transplantation

□ Allogeneic hematopoietic stem cell transplantation

□ Other (__________________)

**2. Cancer recurrence**

**2.1**　Have you ever experienced recurrence of your cancer?

□ Yes

□ No

**2.2**　If **Yes**, what treatment(s) did you receive for recurrent disease? (Select all that apply.)

□ Surgery

□ Systemic drug therapy (including endocrine therapy)

□ Radiation therapy

□ Autologous hematopoietic stem cell transplantation

□ Allogeneic hematopoietic stem cell transplantation

□ Other (__________________)

**3. Current follow-up care**

Which medical institution(s) do you currently attend for cancer follow-up and/or management of treatment-related complications?

Please list **all** institutions if applicable.

**National Cancer Center Hospital**

Department: __________________

Reason for visit: __________________

Department: __________________

Reason for visit: __________________

**Other hospital(s)**

Hospital: __________________

Department: __________________

Reason for visit: __________________

Hospital: __________________

Department: __________________

Reason for visit: __________________

**Reason(s) for current follow-up visits**

□ Cancer surveillance after treatment

□ Endocrine disorders (e.g., short stature, hypothyroidism)

□ Metabolic disorders (e.g., diabetes mellitus, dyslipidemia)

□ Cardiovascular disorders (e.g., hypertension, heart failure, arrhythmia)

□ Kidney disorders

□ Pulmonary disorders

□ Peripheral neuropathy (e.g., numbness or pain)

□ Bone disorders (e.g., osteoporosis)

□ Skin or hair problems

□ Visual problems

□ Hearing problems

□ Problems affecting the hands, feet, bones, or muscles (e.g., prosthetic joints or limbs)

□ Sexual and reproductive health issues (e.g., menstrual disorders, ejaculatory dysfunction, infertility)

□ Lymphedema

□ Difficulties with learning

□ Cognitive problems

□ Mental health concerns (e.g., emotional distress, depression, sleep problems)

□ Other (__________________)

# ***Section III. Long-term Follow-up After Cancer Treatment***

**Definition:** In this questionnaire, long-term follow-up refers to outpatient follow-up visits after completion of routine scheduled cancer surveillance following completion of cancer treatment.

## **1. Long-term follow-up after cancer treatment**

### **1.1 Necessity of long-term follow-up**

Do you think long-term follow-up is necessary after routine scheduled cancer surveillance has ended?
Please select one answer.

□ Strongly agree

□ Agree

□ Neither agree nor disagree

□ Disagree

□ Strongly disagree

### **1.2 Explanation from your doctor**

Did your doctor explain the need for long-term follow-up after routine scheduled cancer surveillance?

□ Yes

□ No

□ I do not remember

### **1.3 Reasons long-term follow-up is necessary**

Why do you think long-term follow-up is necessary? (Select all that apply.)

□ To monitor for cancer recurrence

□ Because treatment-related health problems (late effects) may develop in the future, even if I have no symptoms now

□ To manage existing treatment-related complications

□ To receive updated information about my cancer

□ Other (__________________)

### **1.4 Duration of long-term follow-up**

For how long do you think long-term follow-up should continue?
Please select one answer.

□ 1–2 years

□ 2–5 years

□ 5–10 years

□ As long as possible

□ Until my doctor recommends stopping follow-up

### **1.5 Preferred location for long-term follow-up**

Where would you prefer to receive long-term follow-up care?
Please select one answer.

□ National Cancer Center Hospital

□ A hospital near my home

□ A hospital near my workplace or school

□ Other (__________________)

## **2. Preferred method of follow-up**

### **2.1 Acceptability of different follow-up methods**

Definition: "Online consultation" refers to consultation regarding whether medical attention is required and which department should be consulted. It does not include medical examination or prescription of medications.

| Method | Strongly agree | Agree | Neither agree nor disagree | Disagree | Strongly disagree |
| --- | --- | --- | --- | --- | --- |
| Face-to-face outpatient visits | □ | □ | □ | □ | □ |
| Telemedicine consultation with a physician (including symptom assessment and prescription of medications) | □ | □ | □ | □ | □ |
| Online consultation with a physician (e.g., advice on whether medical evaluation is needed and which department to visit) | □ | □ | □ | □ | □ |
| Online consultation with a healthcare professional other than a physician (e.g., consultation with a nurse before seeing a physician) | □ | □ | □ | □ | □ |

Other: ___________________________

### **2.2 Factors that may make long-term follow-up difficult**

If it became difficult for you to continue attending long-term follow-up visits, what factors might contribute to this?

| Method | Strongly agree | Agree | Neither agree nor disagree | Disagree | Strongly disagree |
| --- | --- | --- | --- | --- | --- |
| I am too busy with work or school. | □ | □ | □ | □ | □ |
| I am too busy with childcare or household responsibilities. | □ | □ | □ | □ | □ |
| I might discontinue follow-up if I moved to another area. | □ | □ | □ | □ | □ |
| I do not want other people to know that I am attending follow-up visits. | □ | □ | □ | □ | □ |
| I might no longer feel that follow-up is necessary. | □ | □ | □ | □ | □ |
| The cost of attending follow-up visits would become a financial burden. | □ | □ | □ | □ | □ |

Other: ___________________________

### **2.3 Current concerns regarding follow-up**

If you have any concerns or difficulties regarding your current follow-up visits, please describe them below.

Your responses are confidential and will not be shared with your treating physician.

____________________________________________________________________

____________________________________________________________________

# ***Section IV. Online Medical Care and Online Consultation***

## **1. Concerns about online medical care and consultation**

### 1.1　**If you were to receive medical care or consultation online, what concerns would you have?**

**(Select all that apply.)**

□ I may have difficulty using a smartphone, computer, or internet connection.

□ I am concerned that communication with healthcare professionals may not be effective.

□ I would be concerned because no physical examination can be performed.

□ Other (________________________________)

## **2. Preferred method of online consultation**

### 2.1　**Which method would you prefer for receiving online medical care or consultation?**

**Please select one answer.**

□ Video consultation using a device with a webcam (e.g., computer, tablet, or smartphone)

□ Telephone

□ E-mail (for online consultation)

□ Messaging application (e.g., LINE)

□ Other (________________________________)

## **3. Preference for replacing current follow-up with online care**

### 3.1　**If your current follow-up visits could be replaced by online medical care, would you prefer this option?**

□ Yes

□ No, I would prefer to continue in-person follow-up visits.

□ I might prefer online medical care if my circumstances change (e.g., relocation or employment).

## **4. Preferred healthcare professional for online consultation**

### 4.1　**If online consultation (other than an online medical visit) were available, whom would you prefer to consult?**

**(Select all that apply.)**

□ Physician

□ Nurse

□ Pharmacist

□ Physical, occupational, or speech therapist

□ Psychologist

□ Medical social worker

□ Registered dietitian

□ Other (________________________________)

## **5. Topics you would like to discuss through online consultation**

### **5.1 Physical concerns**

**(Select all that apply.)**

□ Appearance (physical appearance)

□ Bathing or personal care

□ Breathing problems

□ Urinary problems

□ Constipation

□ Diarrhea

□ Eating or nutrition

□ Indigestion

□ Fatigue

□ Swelling (edema)

□ Fever

□ Activities of daily living

□ Memory or concentration

□ Mouth pain

□ Nausea

□ Nasal dryness or congestion

□ Pain

□ Sexual health

□ Dry or itchy skin

□ Sleep problems

□ Numbness or tingling in the hands or feet

□ Use of over-the-counter medications or supplements

### **5.2 Family-related concerns**

**(Select all that apply.)**

□ Relationship with parents

□ Relationship with children

□ Relationship with partner/spouse

□ Relationship with other family members

□ Fertility / possibility of having children

□ Physical or mental health of family members

### **5.3 Daily life concerns**

**(Select all that apply.)**

□ Financial issues (medical expenses, living expenses, insurance)

□ Transportation (hospital visits, commuting to work or school)

□ Work or school

□ Treatment options

□ Information about disease or treatment

□ Availability of someone to consult

□ Important life events

□ Communication with healthcare professionals

□ Relationships with people other than family

□ Hospitalization

□ Childcare

□ Housework

### **5.4 Emotional concerns**

**(Select all that apply.)**

□ Feeling depressed

□ Anxiety

□ Irritability

□ Fear

□ Tension

□ Sadness

□ Worry

□ Loss of interest in usual activities

### **5.5 Spiritual or religious concerns**

□ Yes

□ No

# ***Section V. Additional Comments Regarding Long-term Follow-up***

Please use the space below to provide any additional comments or suggestions regarding long-term follow-up after cancer treatment.

____________________________________________________________________

____________________________________________________________________

Thank you very much for completing this questionnaire.
