# Supplementary material for: Barriers to survivorship care for adolescent and young adult cancer survivors: perspectives of survivors and cancer specialists
Source: Front Oncol. 2026 Oct 14;16:1977660. doi: 10.3389/fonc.2026.1977660 (PMC13713312; doi:10.3389/fonc.2026.1977660)
Supplement: Supplementary file 2 [file DataSheet2.docx]

**Supplementary Appendix 2**

## **English Translation of the Physician Questionnaire on Long-term Follow-up for Adolescent and Young Adult (AYA) Cancer Survivors**

# ***Section I. Clinical Department***

### 1. Please indicate your clinical department.

Select **one** answer.

□ Neurosurgery and Neuro-Oncology

□ Medical Oncology

□ Gynecology

□ Hematopoietic Stem Cell Transplantation

□ Breast Surgery

□ Urology

□ Musculoskeletal Oncology and Rehabilitation

□ Hematology

□ Pediatric Oncology

□ Radiation Oncology

# ***Section II. Board Certification***

### **1. Please indicate your board certification(s).**

**(Select all that apply.)**

□ Board-certified Neurosurgeon

□ Board-certified Internist

□ Board-certified Medical Oncologist

□ Board-certified Surgeon

□ Board-certified Obstetrician and Gynecologist

□ Board-certified Gynecologic Oncologist

□ Board-certified Breast Surgeon

□ Board-certified Urologist

□ Board-certified Orthopedic Surgeon

□ Board-certified Hematologist

□ Board-certified Pediatrician

□ Board-certified Radiation Oncologist

□ None

# ***Section III. Years of Clinical Experience***

### **1. How many years have you practiced as a physician?**

Please select **one** answer.

□ 5–10 years

□ 10–15 years

□ 15–20 years

□ More than 20 years

# ***Section IV. Long-term Follow-up After Curative Cancer Treatment***

In this section, **long-term follow-up** refers to follow-up care provided **more than five years after completion of curative cancer treatment.** Unless otherwise specified, the following questions refer to **all cancer survivors, including AYA patients.**

## **4a. Departmental policy regarding long-term follow-up**

Which of the following best describes your department’s policy regarding long-term follow-up?

Select **one** answer.

□ A long-term follow-up policy has been established for all patients.

□ A long-term follow-up policy has been established for selected patients.

□ No long-term follow-up policy has been established.

□ Our department does not provide long-term follow-up.

□ Other (__________________)

## **4b. Medical institution responsible for long-term follow-up**

Which medical institution is primarily responsible for providing long-term follow-up?

Select **one** answer.

□ Long-term follow-up is generally provided at our hospital (except for patients who relocate).

□ Some patients are referred to another hospital for long-term follow-up.

□ All patients are referred to another hospital for long-term follow-up.

□ Our department does not provide long-term follow-up.

□ Other (__________________)

### **For respondents who selected: “Long-term follow-up is generally provided at our hospital.”**

## **4b-1. Why is long-term follow-up generally provided at your hospital?**

**(Select all that apply.)**

□ Long-term cancer surveillance is required because of a high risk of recurrence.

□ Long-term health management is required (e.g., monitoring growth and development after childhood or adolescent cancer).

□ Long-term follow-up is required for treatment-related complications or toxicities.

□ There are no suitable hospitals available for referral.

□ Other (__________________)

### **For respondents who selected: “Some patients are referred to another hospital.”**

## **4b-2. Which patients require long-term follow-up at your hospital?**

**(Select all that apply.)**

□ Patients requiring long-term cancer surveillance because of a high risk of recurrence.

□ Patients requiring long-term health management (e.g., monitoring growth and development).

□ Patients requiring long-term follow-up for treatment-related complications or toxicities.

□ Patients who wish to continue follow-up at our hospital.

□ Patients for whom no appropriate referral hospital is available.

□ Other (__________________)

## **4c. Ideal policy for long-term follow-up**

**In your opinion, which of the following statements are appropriate?**

**(Select all that apply.)**

□ Patients at high risk of recurrence should receive long-term follow-up at the hospital where cancer treatment was provided.

□ Patients requiring long-term monitoring of growth and development should receive long-term follow-up at the treating hospital.

□ Patients requiring follow-up for treatment-related complications or toxicities should receive long-term follow-up at the treating hospital.

□ Patients at high risk of late effects should receive long-term follow-up at the treating hospital.

□ AYA cancer survivors should receive long-term follow-up at the treating hospital.

□ All cancer survivors should receive long-term follow-up at the treating hospital.

□ All cancer survivors should be referred to medical institutions capable of providing long-term follow-up.

□ Other (__________________)

# ***Section V. Long-term Follow-up of AYA Cancer Survivors***

## **5a. Challenges in providing follow-up care for AYA cancer survivors**

**Which of the following statements reflect your experience in providing follow-up care for AYA cancer survivors?**

**(Select all that apply.)**

□ Specialized knowledge of fertility preservation and reproductive health is required.

□ Experience in counseling patients regarding life events (e.g., education, employment, marriage, and family planning) is necessary.

□ Scheduling follow-up visits is often difficult because of patients’ school or work commitments.

□ Identifying an appropriate referral hospital for long-term follow-up is often difficult.

□ Follow-up care for AYA cancer survivors is not substantially different from that for adult cancer survivors aged 40 years or older.

□ I have no experience caring for AYA cancer survivors.

□ Other (__________________)

## **5b. Areas in which you feel additional support is needed for long-term health management of AYA cancer survivors**

**For which of the following areas do you feel that management by another specialty or healthcare professional would be necessary?**

**(Select all that apply.)**

□ I have no experience caring for AYA cancer survivors.

□ I feel unable to manage all of the following conditions.

□ Endocrine disorders (e.g., short stature, hypothyroidism)

□ Metabolic disorders (e.g., diabetes mellitus, dyslipidemia)

□ Cardiovascular disorders (e.g., hypertension, heart failure, arrhythmia)

□ Kidney disorders

□ Pulmonary disorders

□ Bone disorders (e.g., osteoporosis)

□ Skin and hair problems

□ Sexual and reproductive health issues (e.g., menstrual disorders, ejaculatory dysfunction, infertility)

□ Lymphedema

□ Cognitive problems

□ Mental health concerns (e.g., emotional distress, depression, sleep problems)

□ Other (__________________)

## **5c. What do you consider to be the major needs for improving follow-up care for AYA cancer survivors?**

**(Select all that apply.)**

□ Clinical support for healthcare professionals providing follow-up care for AYA cancer survivors

□ Clinical guidelines or guidance for long-term follow-up of AYA cancer survivors

□ Information on hospitals and clinical departments capable of providing long-term follow-up for AYA cancer survivors

□ I do not perceive any major challenges.

□ Other (__________________)

***Section VI. Telemedicine for Long-term Follow-up***

**Please provide your comments or suggestions regarding the implementation of telemedicine for long-term follow-up care.**

## **Comments (free text)**

____________________________________________________________________

____________________________________________________________________

Thank you very much for completing this questionnaire.
