# Supplementary material for: Barriers to survivorship care for adolescent and young adult cancer survivors: perspectives of survivors and cancer specialists
Source: Front Oncol. 2026 Oct 14;16:1977660. doi: 10.3389/fonc.2026.1977660 (PMC13713312; doi:10.3389/fonc.2026.1977660)
Supplement: Supplementary file 3 [file Table1.docx]

**Supplemental Table 1. Survivor characteristics (N = 98)**

|  | **N** | **%** |
| --- | --- | --- |
| Age at time of response  　　Teens  20s  30s | 7  42  45 | 7.1  42.9  45.9 |
| Age at diagnosis of cancer  　　Under 10 years old  Teens  20s  30s | 5  24  37  27 | 5.1  24.5  37.8  27.6 |
| Sex  　Male  　Female | 49  47 | 50.0  48.0 |
| Time to hospital  < 30 min  30-60 min  60-120 min  > 120 min | 14  49  27  1 | 14.3  50.0  27.6  1.0 |
| Work or Employment Status at time of response  Regular employee  Non-regular employee  Self-employed  Househusband/Housewife  Student  Unemployed | 48  11  5  8  17  3 | 49.0  11.2  5.1  8.2  17.3  3.1 |
| Prior cancer treatment  Chemotherapy  Surgery  Radiotherapy  Hematopoietic stem cell transplant | 78  40  29  6 | 80.0  40.8  29.6  6.1 |

N: number

**Supplemental Table 2. Cancer specialist characteristics (N = 45)**

|  | **N** | **%** |
| --- | --- | --- |
| Years of experience as a physician  5 to 10 years  10 to 15 years  15 to 20 years  Over 20 years | 1  15  12  17 | 2  33  27  38 |
| Affiliation Department  Brain and spinal cord oncology  Medical oncology  Gynecologic oncology  Hematopoietic stem cell transplantation  Breast surgery  Urologic and retroperitoneal oncology  Bone and soft tissue oncology and rehabilitation  Hematology oncology  Pediatric oncology  Radiation therapy | 3  9  4  3  2  5  5  3  4  8 | 7  20  9  7  4  11  11  7  9  18 |

N: number

One respondent reported affiliation with both Medical Oncology and Urology/Retroperitoneal Oncology; therefore, department-specific affiliations sum to 46 among 45 unique respondents.
